# Morphological variation in paediatric lower limb bones

**DOI:** 10.1038/s41598-022-07267-4

**Published:** 2022-02-28

**Authors:** Laura Carman, Thor F. Besier, Julie Choisne

**Affiliations:** 1 Auckland Bioengineering Institute, The University of Auckland, Auckland, New Zealand; 2 Department of Engineering Science, The University of Auckland, Auckland, New Zealand

**Keywords:** Computational biology and bioinformatics, Ageing, Bone

## Abstract

Available methods for generating paediatric musculoskeletal geometry are to scale generic adult geometry, which is widely accessible but can be inaccurate, or to obtain geometry from medical imaging, which is accurate but time-consuming and costly. A population-based shape model is required to generate accurate and accessible musculoskeletal geometry in a paediatric population. The pelvis, femur, and tibia/fibula were segmented from 333 CT scans of children aged 4–18 years. Bone morphology variation was captured using principal component analysis (PCA). Subsequently, a shape model was developed to predict bone geometry from demographic and linear bone measurements and validated using a leave one out analysis. The shape model was compared to linear scaling of adult and paediatric bone geometry. The PCA captured growth-related changes in bone geometry. The shape model predicted bone geometry with root mean squared error (RMSE) of 2.91 ± 0.99 mm in the pelvis, 2.01 ± 0.62 mm in the femur, and 1.85 ± 0.54 mm in the tibia/fibula. Linear scaling of an adult mesh produced RMSE of 4.79 ± 1.39 mm in the pelvis, 4.38 ± 0.72 mm in the femur, and 4.39 ± 0.86 mm in the tibia/fibula. We have developed a method for capturing and predicting lower limb bone shape variation in a paediatric population more accurately than linear scaling without using medical imaging.

## Introduction

Childhood conditions such as cerebral palsy, developmental hip dysplasia, and slipped capital femoral epiphysis have a negative effect on lower limb mobility^1^. Personalised musculoskeletal modelling can help to understand structure–function relationships in both typically developed (TD) children and children affected by movement disorders^2^. Three-dimensional gait analysis, for example, can be used in conjunction with musculoskeletal models to understand muscle–tendon function and predict outcomes from surgical intervention^3^. Computational models have the potential to assist surgeons to deliver appropriate interventions, customise orthoses, and monitor patient rehabilitation. However, the first step in generating a personalised musculoskeletal model is to accurately capture the anatomy of the participant, which is often the most labour-intensive and challenging.

A common method for generating musculoskeletal models is to scale the bone and muscle geometry from an existing adult template^4^. Scaling is performed either by using relative measurements of anatomical landmarks (typically segment lengths calculated from optical motion capture markers) or by height and mass. Scaling might work when the template model is of similar proportions to the target participant. However, linear scaling of body segments is unlikely to accurately capture the geometry of a paediatric population, as children are not just scaled down adults; they differ significantly in geometry and physiology^5–7^. To generate personalised paediatric musculoskeletal models, we need representative template models from a paediatric population. An accurate but time-consuming approach is to create subject-specific models from medical images, such as MRI or CT, which in children can take 10–12 h per case^5^. Subject-specific models produce different results compared to generically scaled models in both adults and children^2,8–11^. We propose an alternative approach to create paediatric musculoskeletal models using a statistical shape model (herein referred to as a ‘shape model’) to scale a template model to match a participant’s anthropometry. The template model represents the mean paediatric bone geometry in the population. This approach bridges a gap between generic and patient-specific models, providing easy and rapid generation of anatomically accurate models.

Shape models typically use a principal component analysis (PCA) to characterise anatomical variation as a combination of weights and principal components, or modes. This approach has been used to capture the geometry of the adult pelvis^12,13^, femur^14–20^, the knee joint^21^, the tibia^20^, and the lower limbs collectively^22^. Using a shape model of the adult femur, for example, geometry can be predicted within 2.3 mm root mean square error using just six parameters: age, sex, height, body mass, femoral length, and epicondylar width^14^. To our knowledge, existing studies of this type on the lower limb have only been conducted on adult cohorts and adult shape models do not accurately predict paediatric bone^5^.

The development of a paediatric shape model to predict musculoskeletal geometry in children could provide a solution for modelling the lower limbs in a paediatric population. Such a shape model will likely produce more accurate results than a linearly scaled generic model as has been seen in adults^22,23^ and is more reasonable for widespread implementation than a model created from medical imaging. Creating a population-based model from a large dataset of bones will allow us to analyse and predict changes in musculoskeletal geometry, as has been done in adult populations^22^. This will allow the observation of how musculoskeletal shape changes with growth on a scale which has not been seen in the paediatric population. These valuable data will help to inform clinical decisions and form a comparison for children with movement abnormalities.

The objectives of this research were to: (1) understand how bone shape differs within a typically developed paediatric cohort aged from 4 to 18 years, (2) evaluate the accuracy of bone shape prediction in the lower limbs and determine the factors which affect bone shape, and (3) evaluate the differences between shape model predicted bone geometry and linearly scaled bone geometry.

## Methods

### Bone segmentation

Retrospective post-mortem CT scans of 333 children (137 F, age: 4–18 years (12 ± 5 Y), Height: 96–192 cm (148 ± 24 cm), Mass: 14–140 kg (49 ± 22 kg)) were obtained from the Victorian Institute of Forensic Medicine (VIFM, Melbourne, Australia) with ethics approval EC 22/2016 from the VIFM Ethics Committee. This study used retrospective data, which was collected by the VIFM for autopsy purposes between 2006 and 2019. Before autopsy, the VIFM obtained written consent from the individual’s legal guardian. All methods were performed in accordance with the relevant guidelines and regulations. The resolution and slice thickness varied between cases, as the scans were taken over a period of 14 years (2006–2019). The slice thickness of the images ranged from 0.5 to 2 mm and the pixel spacing from 0.57 × 0.57 to 1.27 × 1.27 mm. Any participants with ailments affecting the lower limb structure and function were removed from the dataset.

The bones segmented included the pelvis, femurs, tibias, and fibulas. Manual segmentation on Mimics Research Suite v23 (Materialise, Leuven, BE) was performed for 146 of these cases, the rest were automatically segmented using a machine learning algorithm (Deep Segmentation, Formus Labs), to spend less time performing manual segmentations. The automatically segmented bones were each visually inspected to check for discontinuities in any areas, then smoothed, and re-meshed using MeshLab^24^ ‘Uniform Mesh Resampling’ and ‘Laplacian Smooth’ functions. Discontinuities included merging of two bones at the joint space and meshing errors such as overlapping faces. Accuracy of the deep segmentation can be found in Supplementary material (Table S1). Out of the 187 automatically segmented cases, 246 bones displayed discontinuities across 72 cases and subsequently were disregarded and manually segmented in Mimics. The areas where the algorithm failed were mostly in the cases of young children, with 40 of the failed cases being below 10 years old and 25 of the failed cases being 4–5 years old. This was likely due to the prominent growth plate seen in young children making it difficult for the algorithm to determine where the bone begins and ends. The bone type with the highest fail rate was the femur, accounting for 86 of the failed bones.

After all bones were segmented from the CT scans, all right bones from both segmentation pathways were mirrored to the left side for the femur, tibia, and fibula to increase the sample size for the shape model and to only require a single shape model for the femur and tibia/fibula. The tibia and fibula were combined (tibia/fibula). The total number of bones for the pelvis was 331, for the femur was 663, and for the tibia/fibula was 658. Next, all bones were aligned in accordance with their individual centre of mass based on anatomical landmarks located on each bone.

### Principal component analysis

Principal Component Analysis (PCA) was conducted for each bone in the dataset. PCA is an unsupervised machine learning algorithm used to mathematically describe variation in a dataset using orthogonal projection, called principal components (PCs) or modes^25^. The weights of the PCs can be adjusted to predict new bone geometries outside of the dataset. PCA was performed using GIAS2, an open-source Python library^14^. PCA was performed for the pelvis, femur, and tibia/fibula separately following the workflow outlined below.

**Figure 1:**
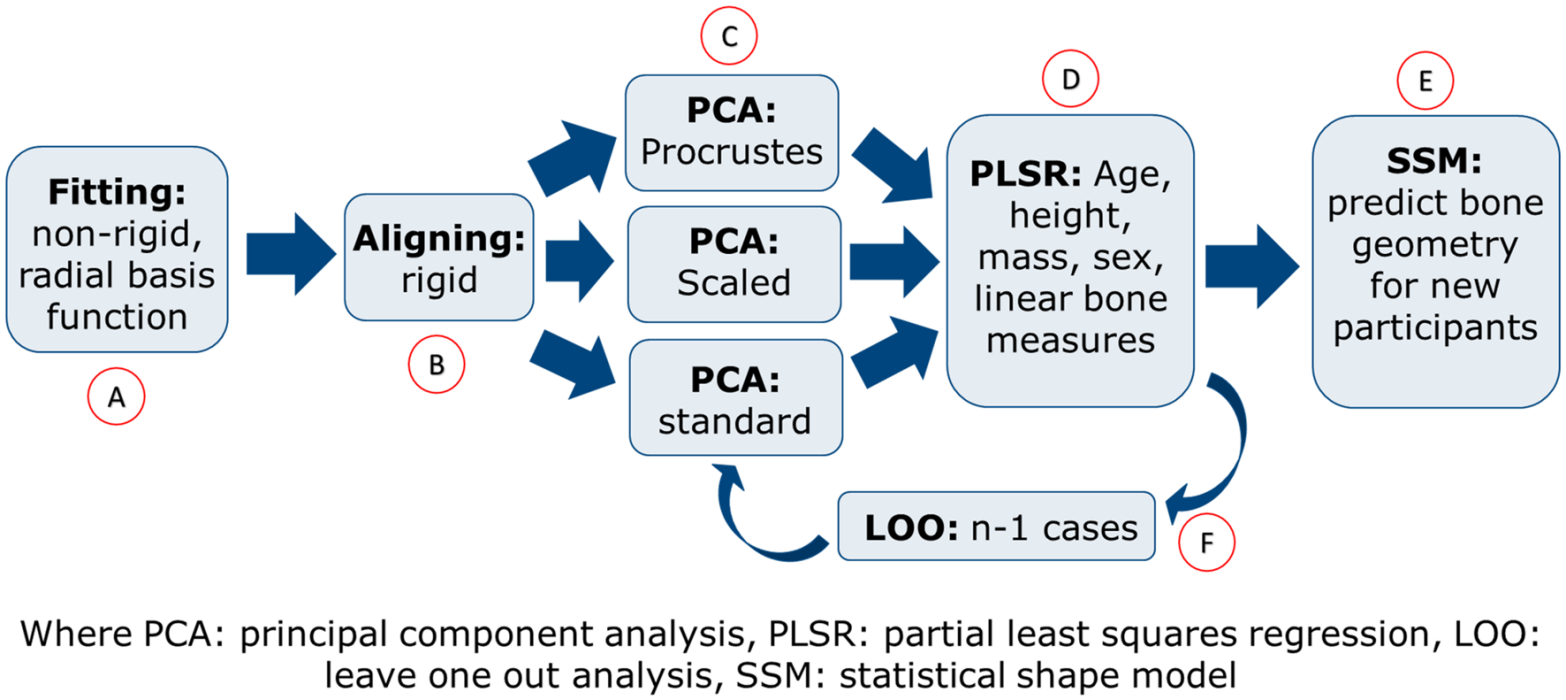
Workflow for the development of PCA (A, B and C) and modelling and testing of the statistical shape model (D, E and F).

1. *Fitting (Fig. **1**A)* The first step in the workflow was to non-rigidly register, using radial basis functions, each of the bone meshes in the dataset to the template mesh to achieve nodal correspondence between the meshes. The template mesh is chosen as the mesh with the desired number of nodes and node distributions for which all meshes in the dataset will be fitted to. The template mesh for the femur and tibia/fibula arose from the same case (F, 5Y, 111 cm, 19 kg) and for the pelvis from another case (M, 8Y, 112 cm, 24 kg) selected due to being able to fit a large range of meshes.
2. *Alignment (Fig. **1**B)* The second step was to rigidly align the fitted meshes to remove rotational and translational variations, by aligning all bones to the template mesh using centre of mass.
3. *Principal component analysis (Fig. **1**C)* The final step was to perform a PCA on the aligned meshes to generate the mean mesh and the principal components of variation in the dataset. If the dataset is large enough, these principal components should capture the variation present in a general paediatric population. This was quantified through percentage variation captured by the model and prediction errors (discussed in “Accuracy of the statistical shape models” section).

### Standard shape model

For the standard shape model, a PCA was performed with all bones at their original size to retain both size and shape variation for the pelvis, femur and tibia/fibula.

### Procrustes analysis

A Procrustes analysis was performed to remove the size variation between participants and therefore have a better understanding of the shape variation of the bones using the GIAS2 library^14^. This involved scaling all bones to the size (volume) of the mean bone shape and performing PCA on these scaled meshes (Fig. 1C). This enabled the analysis of shape variation independent of size, which was a dominant component due to the age range in this dataset.

### Statistical shape modelling

The PC weights from the PCA were used to train a Partial Least Squares Regression (PLSR) (Fig. 1D) which was subsequently used to predict bone geometry. The predictive factors consisted of demographic factors and linear bone measurements. The demographic factors were; age, height, mass, and sex. The linear bone measurements included: width between anterior superior iliac spines and width between posterior superior iliac spines (pelvis); epicondylar width and femur length (femur); tibial condyle width, malleolar width, and tibial length (tibia and fibula). These predictor variables were chosen based on their ability to be easily measured in a clinic, without the need for medical imaging. PLSR was used to determine the individual weighting for each PC for the bones to be predicted relative to the weightings of the training dataset. The personalised surface bone geometry was generated from these weightings and the mean mesh from the PCA (refer to “Principal component analysis” section) (Fig. 1E).

### Scaled shape model

A second shape model was created to remove the large influence of size from the PCA. This was done by uniformly scaling all the bones in the dataset by 100/x where x was the length measurement for each bone (ASIS width, femoral length, and tibial length). PCA was then performed on these length-scaled bones. Subsequently, when these bones were predicted by the shape model, they were scaled back to their original size by x/100. This shape model required the additional knowledge of bone length measurements to reconstruct the bone morphology.

### Accuracy of the statistical shape models

To quantify the accuracy of our shape model, we calculated the following metrics, as suggested by Ref.^26^.

#### Fitting error

Measures the surface distance between the non-rigidly registered fitted mesh and the originally segmented mesh. This was conducted using the cloud-to-mesh distance function on CloudCompare^27^, which provides the node-to-surface distance for each node on the originally segmented mesh. The root mean squared error (RMSE) was then calculated using these distances.

#### Compactness

Measures the ability of the model to accurately capture the variation in the data while using the minimum number of principal components. This was assessed by iteratively increasing the number of principal components used in the shape model to predict bone geometry. Then comparing mesh distances between the predicted mesh and the corresponding segmented and aligned mesh. The number of PCs was increased to n−1 (where n = number of bones) and a testing set of 10% of the dataset was used for prediction in the interest of computational time.

#### Specificity/prediction error

Measures the ability of the model to predict bone shapes within the dataset. This was performed using a number of principal components determined by the compactness analysis, to predict each case in the training set based on the weighting output by the PCA. For each case the corresponding principal component weightings were input into the shape model and a predicted mesh was calculated. The predicted mesh was compared to the corresponding segmented and aligned mesh using node to node distances and RMSE was then calculated using these distances.

#### Generality

Measures the ability of the model to predict bone shapes which are outside of the dataset. This was performed using a leave one out analysis (LOO), where we trained the shape model using N−1 participants and predicted the bone geometry of the one ‘left out’ participant (Fig. 1F). Next, predicted meshes were compared to the ground truth (segmented meshes) to assess the accuracy of the prediction. The mesh-to-mesh distances were quantified using RMSE distances, volume differences, and dice scores. This was conducted using a custom python script. To quantify the predictive power of the factors used in the shape model, a multiple comparison analysis was performed to find the coefficient of determination (R^2^) between each principal component and predictive factor. The best predictive factors were also determined for each bone using PLSR between all principal components and different combinations of predictive factors. This was used to determine the relevance of the predictive factor in the shape model and whether it should be included for prediction. LOO analysis was performed for the shape model using the best predictive factors and the best demographic factors and for the scaled shape model using the best predictive factors.

### Comparison to linear scaling

To understand the advantage of using a shape model to predict bone geometry, various types of geometry creation were compared.

1. *Gold-standard geometry* bone geometry segmented from the CT scans.
2. *Shape model predicted geometry* generated from the best predictive shape model for each bone using the best predictive factors.
3. *Linearly scaled mean paediatric geometry* found by using the mean mesh from the standard shape model and linearly scaling this mesh by the relative bone length measurement between the mean mesh and the mesh to be predicted.
4. *Linearly scaled adult geometry* found by using the adult bone geometry from OpenSim^4^ and linearly scaling this mesh by the relative bone length measurement between the adult mesh and the mesh to be predicted.

The gold standard geometry was compared to each of the different predicted meshes using RMSE distances and dice scores. The bone length measurements for the pelvis, femur and tibia/fibula were ASIS width, femoral length, and tibial length respectively.

## Results

### Principal component analysis

The percentage of variation accounted for by the PCA of the pelvis, femur, and tibia/fibula showed most of the variation was captured in the first principal component, accounting for overall size. This was 92% for the pelvis, 98% for the femur, and 97% for the tibia/fibula (refer to supplementary material Fig. S1 for visualisations).

### Procrustes analysis (shape variation only)

**Figure 2:**
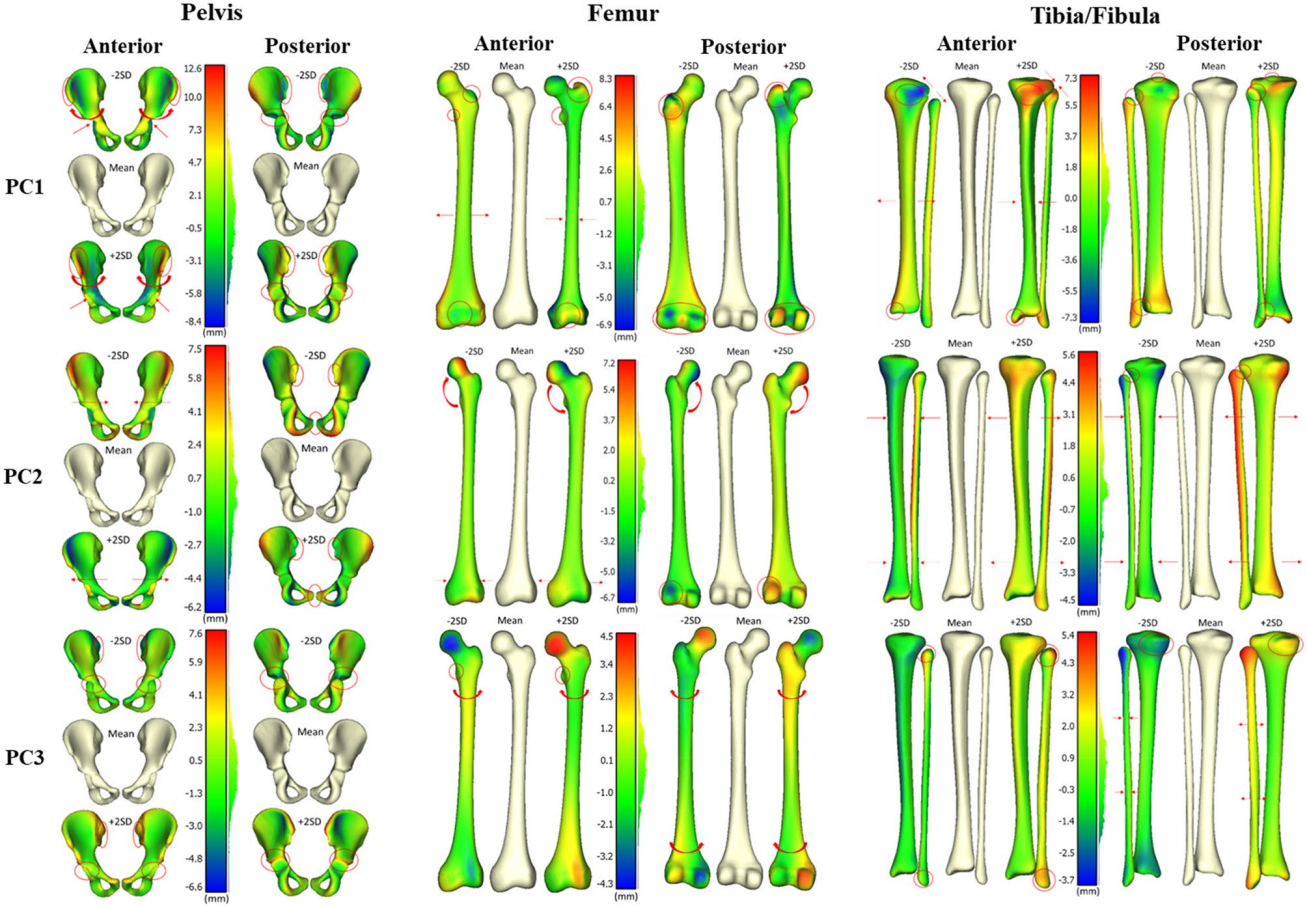
Coloured distance differences of mean meshes (cream) and ± 2SD for first three principal components following Procrustes analysis (i.e. shape variation only). Anterior (left) and posterior (right) viewpoints are shown and red ellipses and red arrows highlight main features of difference to the mean mesh.

The percentage of variation accounted for in the Procrustes PCA for the pelvis, femur, and tibia/fibula showed a reduction in the variation accounted for by the first principal component as size differences were removed (Fig. 2). 90% of the variation was captured in the first 41 PCs for the pelvis, 60 PCs for the femur, and 39 PCs for the tibia/fibula.

#### Pelvis

The first PC represented rotation of the ilia and a subtle change in width at the bottom of the pelvis. Shape changes in the acetabulum were also captured by the first PC. The second PC represented changes in the width of the pelvis, changes in the pubic arches and the definition of the PSIS’. The third PC represented inward growth in the PSIS’ and formation of the acetabular socket (Fig. 2).

#### Femur

The first PC captured variations in the greater and lesser trochanter, epicondylar width, diaphysis width and definition of the condyles. The second PC represented the variation in width of the femur and the change in neck shaft angle. The third PC captured femoral anteversion and definition of the lesser trochanter (Fig. 2).

#### Tibia/fibula

The first PC represented the width of the tibia/fibula, the definition of the tibial tuberosity, intercondylar tibial tubercles, the fibular notch, the proximal tibiofibular joint, the formation of the medial malleolus, and growth changes at the epiphyses. The second PC of tibia/fibula captured the space between the tibia and fibula, and definition of the proximal tibiofibular joint. The third PC of the tibia/fibula represented the width of the fibula, the size of the head of the fibula and lateral malleolus, and definition of the tibia condyles (Fig. 2).

### Scaled model analysis

The percentage of variation accounted for in the scaled PCA for the pelvis, femur, and tibia/fibula showed a reduction in the variation accounted for by the first PC compared to the standard PCA. 90% of the variation was captured in the first 9 PCs for the pelvis, 36 PCs for the femur, and 15 PCs for the tibia/fibula.

### Accuracy of the shape models

#### Fitting and prediction errors

The fitting errors were low for all bones, for the pelvis this was 0.35 ± 0.08 mm, the femur 0.20 ± 0.04 mm, and the tibia/fibula 0.16 ± 0.04 mm. The prediction errors for the shape model for the femur, tibia/fibula, and pelvis decreased for increasing PCs for all bones (Fig. S2). The first 100 PCs were used to reconstruct the bones as this provided average RMSE < 1 mm for all bones. Prediction errors in the initial shape model (including size and shape) were similar to the Procrustes model. However, prediction errors in the scaled model were lower by between 0.23 and 0.28 mm (Table 1). Further comparisons were therefore made using this scaled model.

**Table 1:** Prediction errors for the pelvis, femur, and tibia/fibula from the three types of statistical shape model.

	Prediction error standard model (mm)	Prediction error procrustes (mm)	Prediction error scaled model (mm)
Pelvis	0.47 ± 0.05	0.47 ± 0.04	0.24 ± 0.02
Femur	0.38 ± 0.05	0.41 ± 0.04	0.13 ± 0.01
Tibia/fibula	0.41 ± 0.05	0.41 ± 0.04	0.13 ± 0.01

Prediction errors are calculated using the first 100 principal components and displayed as RMSE.

#### Multiple comparison analysis

The multiple comparison analyses between the first principal component and each of the predictive factors for the standard and scaled shape models are shown in Table 2. In the standard shape model the pelvis had high percentage variation explained by all predictive factors besides sex. The femur and tibia/fibula had moderate percentage variation explained by mass and high percentage variation explained by all other factors apart from sex. There were no significant variations explained by any of the predictive factors in the scaled shape model. This may imply the ability for this model to predict new geometries from these predictive factors is limited.

**Table 2:** Multiple comparison analysis results for the standard and scaled shape models showing the R^2^ scores for each predictive factor for the pelvis, femur, and tibia/fibula.

R^2^	Model	Age	Height	Mass	Sex	ASIS width	PSIS width	Epicondylar width	Femoral length	Condyle width	Malleolar width	Tibial length
Pelvis	Standard	**0.881**	**0.926**	**0.737**	0.025	**0.823**	**0.782**					
	Scaled	0.263	0.238	0.132	0.048	0.006	0.277					
Femur	Standard	**0.837**	**0.969**	*0.689*	0.002			**0.895**	**0.995**			
	Scaled	0.072	0.070	0.015	0.087			0.032	0.145			
Tibia/fibula	Standard	**0.805**	**0.966**	*0.689*	0.001					**0.868**	**0.885**	**0.985**
	Scaled	0.237	0.304	0.158	0.006					0.217	0.319	0.441

Bold shows high percentage variation explained by that factor, and italics shows moderate percentage variation explained.

Table 3 demonstrates the best predictive factors for each bone based on the highest R^2^ value for both the standard and scaled shape models. In the standard shape model pelvis shape was best explained by the combination of all predictive factors whereas the femur and tibia/fibula were best explained by height and femoral and tibial length. If length measurements are not available the pelvis shape was best explained by all factors, the femur by age and height, and the tibia/fibula by height and mass. The best predictive factors in the scaled model for the pelvis were age, height, ASIS width, and PSIS width. For the femur these were height, epicondylar width, and femoral length. And for the tibia/fibula these were height and tibial length. Using only demographic measurements for prediction can be disregarded as this model requires length measurements as input for scaling.

**Table 3:** The combination of predictive factors which produced the highest R^2^ score in a partial least squares regression of the standard and scaled shape models.

	Standard model		Scaled model
	Best predictive factors	Best predictive demographic	Best predictive factors
Pelvis	All factors 0.976	All factors 0.951	Age, height, ASIS width, PSIS width 0.825
Femur	Height, femoral length 0.997	Age, height 0.970	Height, epicondylar width, femoral length 0.419
Tibia/fibula	Height, tibial Length 0.990	Height, mass 0.966	Height, tibial length 0.611

Where best predictive factors considered all factors: age, height, mass, sex, and length measurements. Best predictive demographic considered only age, height, mass, and sex for predictive factors.

#### Leave one out analysis

**Figure 3:**
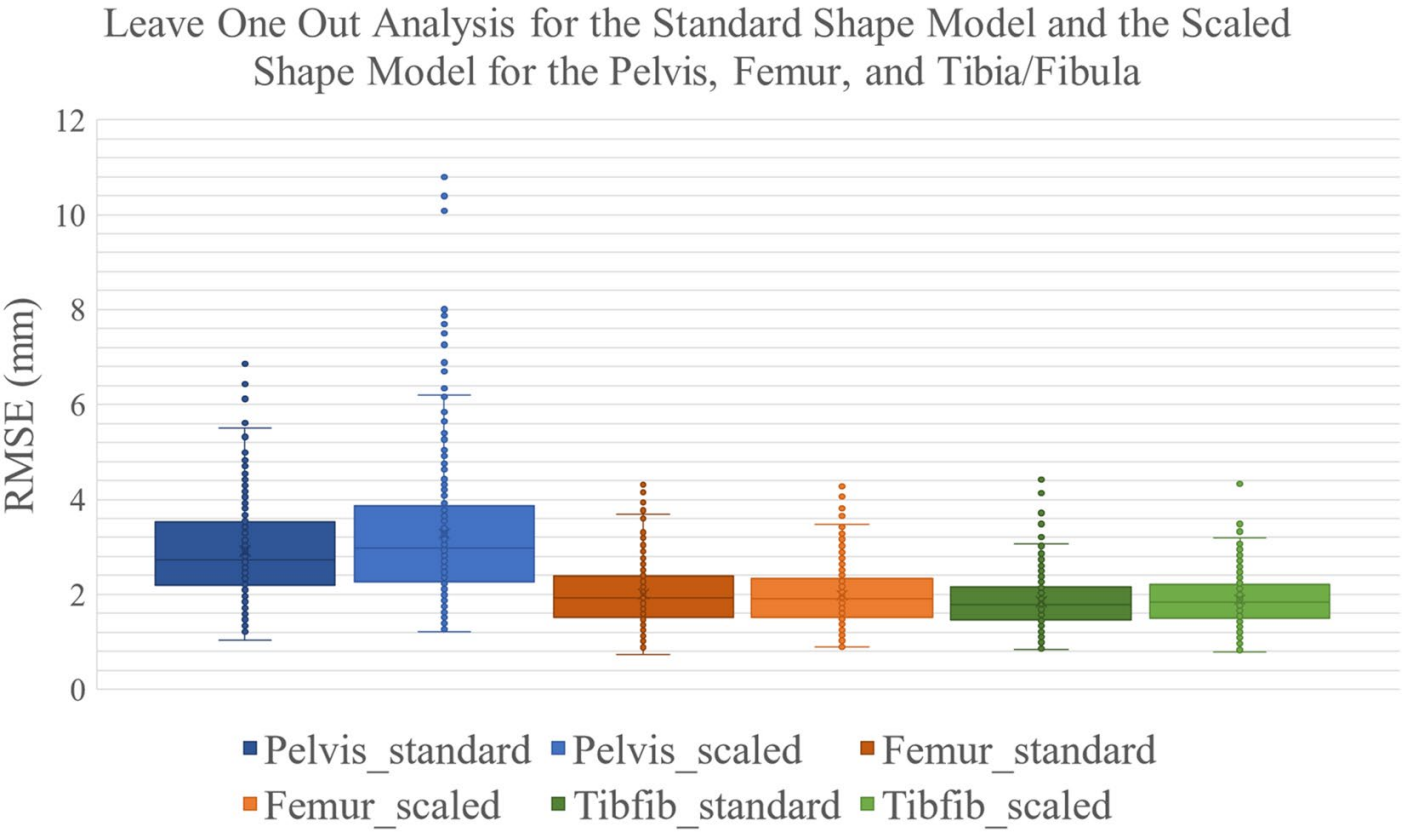
Results from the leave one out analysis of the Pelvis, femur and tibia/fibula (tibfib) for the standard shape model and the scaled shape model (scaled) using all predictive factors.

The RMSE was lower in the standard shape model when length measurements were included, however the errors were still low when only demographic measurements were available (Table 4). When comparing between the standard shape model and the scaled shape model, the scaled shape model showed higher errors in the pelvis and similar errors for the femur and tibia/fibula. The same conclusions were found when looking at the volume errors and the dice scores. Further comparison of the standard shape model to the scaled shape model showed a larger range of values for the pelvis in the scaled model (Fig. 3). For the femur, the two models were similar. In the tibia/fibula, the scaled model gave a slightly lower spread of values. The scaled model had no added benefit over the standard shape model, and performed worse in the pelvis, therefore, the standard shape model was chosen as our final model.

**Table 4:** Results from the leave one out analysis showing the average RMSE (mm) ± 1SD, percentage volume error ± 1SD, and dice score ± 1SD.

	Bone	Standard model all factors	Scaled model all factors	Standard model demographic factors
RMSE (mm)	Pelvis	2.91 ± 0.99	3.28 ± 1.54	3.23 ± 1.22
	Femur	2.01 ± 0.62	1.98 ± 0.61	2.72 ± 1.24
	Tibia/fibula	1.85 ± 0.54	1.89 ± 0.54	2.25 ± 0.96
Volume Error (%)	Pelvis	9.90 ± 8.29	16.62 ± 13.90	10.76 ± 9.18
	Femur	8.62 ± 8.09	8.60 ± 6.60	8.90 ± 9.20
	Tibia/fibula	9.95 ± 9.86	10.07 ± 9.03	11.17 ± 12.47
Dice Score	Pelvis	0.77 ± 0.07	0.75 ± 0.10	0.74 ± 0.09
	Femur	0.89 ± 0.03	0.89 ± 0.03	0.86 ± 0.06
	Tibia/fibula	0.86 ± 0.04	0.86 ± 0.04	0.84 ± 0.05

The errors represent the difference between shape model generated bone geometries and the segmented geometries (gold standard). “All factors” uses the best demographic and length measurements for prediction. Where demographic factors are age, height, mass, and sex. Shown are the results for the standard shape model and the scaled shape model.

### Comparison to linear scaling

**Figure 4:**
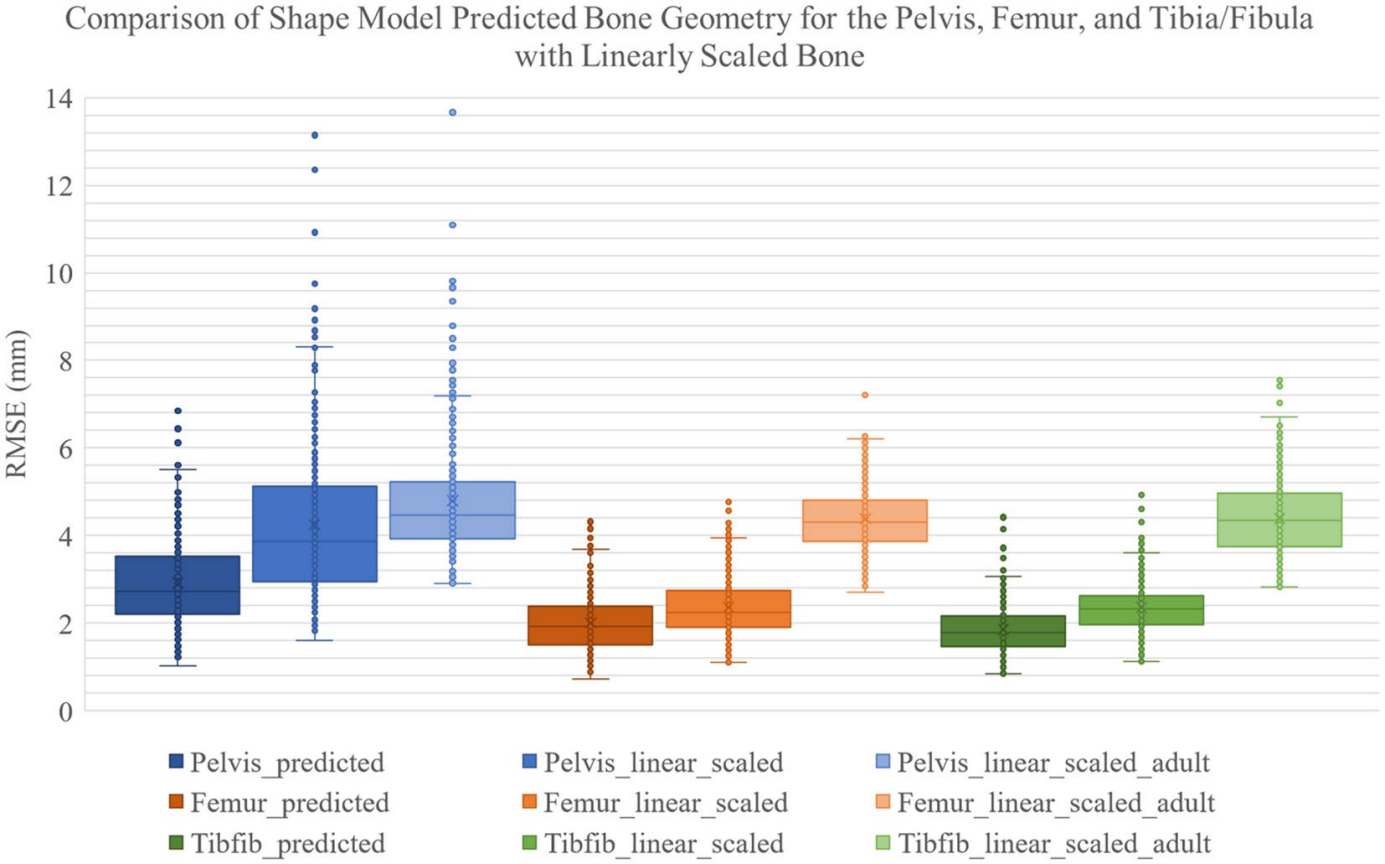
Results for the pelvis, femur, and tibia/fibula (tibfib) showing the RMSE (mm) for bone geometry predicted using the standard shape model (_predicted), linearly scaled geometry from the mean mesh of the shape model (_linear_scaled), and linearly scaled geometry from adult OpenSim geometry (_linear_scaled_adult).

Comparison of the shape model to linear scaling methods showed the advantage of using a shape model over linear scaling. RMSE distances demonstrated that linear scaling using the mean shape model mesh had slightly higher errors on average (< 1 mm) for the femur and tibia/fibula and larger errors for the pelvis (> 1 mm) compared to bone predicted using the shape model. Linear scaling of an adult mesh produced, on average, higher errors compared to the shape model predicted meshes; 1.88 mm in the pelvis, 2.37 mm in the femur, and 2.54 mm in the tibia/fibula. RMSE results are displayed in Fig. 4 and RMSE values and dice scores are shown in supplementary material (Table S2). Dice scores showed similar results with low dice scores in the linearly scaled adult cases (0.56–0.7) compared to shape model predicted (0.77–0.89) and linearly scaling the mean shape model mesh (0.66–0.87). We can see a large shift in the results from the linearly scaled adult mesh for all bones and a smaller shift in the linearly scaled paediatric mesh (Fig. 4). Visualisation of the effects of linear scaling are shown in the supplementary material. Figure S3 shows an average case in the dataset which has lower but visible differences. Figure S4 shows the highest error cases when scaling adult bone geometry, which has marked differences in all bones, especially for the pelvis.

## Discussion

Statistical shape modelling is a common method used to capture bone geometry in an adult population^14,21^, but has not been widely explored in a paediatric population. We presented a statistical shape model of the pelvis, femur, and tibia/fibula surface geometry in a paediatric population. The aims of this study were to understand the differences in bone shape in children, predict unknown bone geometries using clinically available measurements, and compare the performance of the shape model to traditional linear scaling.

The results gathered showed the justification of using a shape model to understand and predict the bone geometry of children. The variation seen in the principal components and the high percentage captured by the first component are indicative of the great range of bone sizes seen in this dataset and capture the variation in bone shape with growth. In this study, the percentage variation captured by the first principal component was above 90% for all bones. Comparing to similar adult studies, this value is around 24% for the pelvis^13^, and 35–45% for the femur^14,18,19^. Other studies in adults found 95% of the variation was found in the first 20 (for the pelvis), 4–10 (for the femur), and 8 (for the tibia) principal components^12,20^. Another PCA created for the tibia captured 96% of variation in the first principal component^28^. This shows a large variation in the percentage between studies, mostly due to the population used and size variation within the dataset. While the percentage variation captured in the first principal component seems too large for the model to capture non-size related changes in the bone, we can see changes in the first principal component other than size, such as the emergence of the greater and lesser trochanters in the femur.

The shape changes captured by the first three principal components are comparable to existing adult studies. For example, in a study of the adult pelvis; PC1 captured changes in size variation, PC2 captured changes in the size of the ilia and pubic arches, and PC3 captured changes in width and rotation of the ilia^13^. This is similar to the current study with PC2 and PC3 swapped. Across various studies in the adult femur; PC1 captured length/scale changes, PC2 captured anteversion angle, neck shaft angle, and width changes, and PC3 captured shaft width, anteversion angle, neck shaft angle, and head diameter changes^14,18,19^. These findings are similar to those obtained in this study. In the adult tibia; PC1 captured length/scale changes, PC2 captured slope changes in the medial tibial plateau and valgus deformities, and PC3 captured bowing of the shaft^28^. We had similar findings, with the addition of width changes and the interaction with the fibula. In general, the principal components obtained in this study were consistent with those obtained in the same bones in adult studies, with the exception of the emergence of growth-related changes, such as the greater trochanter in the femur and the tibial tuberosity, in the paediatric dataset.

The large size variation captured by the first principal component across all bones showed the need for a Procrustes analysis, where the shape variation of the dataset could be viewed by removing size variation to better understand and visualise the changes in bone shape. The Procrustes analysis showed the emergence of different shape variations in each principal component, which were difficult to visualise when size variation was included (Fig. 2). Growth-related changes seen in the pelvis included the spherical formation of the acetabulum, the rounding of the ilia, and the thickening of the pubic arches. The growth-related changes seen in the femur included the formation of the greater and lesser trochanter, and the formation of the femoral condyles. The growth-related changes seen in the tibia/fibula included the formation of the tibial tuberosity, the intercondylar tibial tubercles, the emergence of the medial malleolus, and merging of the tibiofibular joint. The emergence of these features is consistent with knowledge of bone growth^29^. Many growth-related changes occur due to forces exerted on the bone by muscles, which increase during growth^30^. The Procrustes analysis was able to show these potential growth-related changes in the pelvis, femur, and tibia/fibula in a way that has not been seen before in a paediatric population. However, the Procrustes analysis cannot be used to predict unknown bone geometries as the scaling factor is not known. Therefore, the standard and scaled shape models were compared to determine the best predictive model.

The best predictive factors for each bone were similar in the femur and tibia/fibula but different for the pelvis. This is likely due to the complex geometry of the pelvis, which is less influenced by height compared to the long bones. In the case of the long bones, sex was not found to be an important predictive factor, while in the pelvis, sex was shown to be important. For the femur and tibia/fibula height and length measurements were the most important for predicting unknown bone geometries. This indicates that age does not contribute to the prediction of long bone shape as much as height and length. This can be accounted for by considering bone remodels to the strains it experiences (i.e. Wolff’s law); bone length affects the moment arms of the muscles and consequently the torques produced by the musculoskeletal system. Therefore, typically developed children of a similar height and bone lengths are likely to have similar bone morphology, regardless of biological age. This statement may not hold true for children with bony deformities as this study has been conducted on a typically developed population of children. This is not to say that the bone morphology is predicted only by linear scaling, as the statistical nature of the principal component analysis accounts for other variations in the bone beyond simple linear length scaling. In the absence of bone length measurements, height along with age was shown to be an important predictive factor for the femur, and along with mass for the tibia/fibula, showing that age and mass are redundant when length measurements are available.

A leave one out analysis was performed to predict bone shape using only demographic and length measurements. The results of this analysis showed low prediction errors across all bones, with average RMSE of 2.91 mm in the pelvis, 2.01 mm in the femur, and 1.85 mm in the tibia/fibula. Comparing to a similar study on the adult femur, bone shape was predicted with an RMS error of 2.3 mm using the same demographic and linear bone measurements, however, the best predictive factors were not determined in that study^14^. Other studies have predicted bone geometry using anatomical landmarks, finding errors in the pelvis of 4.23–5.4 mm, the femur of 2.6–4.8 mm, and the tibia/fibula of 2.88–3.63 mm^12,22,23^. Each study used different bone landmarks, which in some cases are only available using medical imaging. Most of these studies had a small sample size in comparison to the current study, which may have led to greater errors. Our errors fall within a similar range compared to existing adult studies for bone shape prediction from clinical measurements.

The leave one out analysis of the shape model has some cases of higher error than others, these cases were found to be shape outliers in the dataset when compared to other cases of similar predictive factor values. These cases were minimal but will be improved upon when using an articulated shape model, which will be informed by marker data and medical imaging and therefore should be more likely to capture these outliers.

The scaled shape model was developed to understand if including the size variation in the shape model affected the prediction of new bone geometries. Using a scaled shape model provided similar performance in the femur and tibia/fibula but higher errors in the pelvis. Therefore, this model did not provide additional benefit for bone prediction, compared to the non-scaled standard model. This model also required linear length measurements, which may not always be available clinically and is more time-consuming to implement. The standard shape model was chosen as the final model for this dataset.

Investigating effects of linear scaling on bone geometry showed that by linearly scaling the average paediatric mesh from the shape model produced slightly less accurate results than using the shape model predicted bone geometry. However, the average paediatric mesh (informed by the current shape model and population data) is not currently available to the wider community. To compare current scaling methods for bone geometry in children, adult bone geometry^4^ was linearly scaled, which produced less accurate predictions of all bones. This shows the advantage of using a statistical shape model to predict bone geometry in a paediatric population when medical imaging is unavailable. Additionally, linear scaling had a greater effect on the accuracy of the pelvis than the femur and tibia/fibula. This is due to the more complex geometry of the pelvis and shows the need to predict the pelvis rather than scale the pelvis to obtain more accurate hip joint centre locations^31^. Similar studies have examined the effect of linear scaling on adult bones finding differences in the errors between shape model predicted bone and linear scaling when comparing to the gold-standard model segmented from medical imaging. Average differences in the pelvis of 1.23 mm, femur of 0.84–1.06 mm, and tibia/fibula of 0.04–0.99 mm were found^22,23^. In a paediatric population RMSE from linear scaling were found to be 12.57 mm in the pelvis, 7.43 mm in the femur, and 8.16 mm in the tibia/fibula^5^. These errors are larger than obtained in this study and may be due to our dataset being much larger. This shows that the effects of linear scaling of adult bone in a paediatric population is much greater than linear scaling adult bone, highlighting the importance of a musculoskeletal atlas for a paediatric population which can predict bone geometry without the need for medical imaging.

Limitations in the current study include bone measurements being taken from the bone surface rather than the skin surface. Real world measurements might include soft tissue artefact when estimating anatomical landmarks, which might lead to less accurate predictions. The dataset was obtained from an urban Australian population and therefore, the shape model may not represent a global or specific indigenous population. Additionally, some age groups had fewer number of CT scans available (e.g. 7 to 10 years old) meaning these age groups may not yet be fully represented. Inferences regarding morphological bone changes due to growth should be made with caution, as these data are cross-sectional, rather than longitudinal. Creation of lower limb musculoskeletal models also requires addition of the patellae and feet, which were not included in this study and could limit accuracy of future models. This was due to the patella not being formed yet in many young children and the feet being cut off in many of the CT scans. The shape models generated here were created for each bone independently, ignoring structure–function relationships that could improve the prediction of bone morphology across multiple bones. An articulated shape model that includes pose as well as combined bone morphology could improve the robustness of bone morphology prediction, as demonstrated by Zhang et al.^22^ and will be the focus of future work. As will be the integration of this model into the opensource software MAP client^32^ to provide the ability to create musculoskeletal models in a paediatric population. The articulated shape model will include prediction of muscle attachment sites for use in musculoskeletal modelling software. More paediatric MRI scans will be obtained in longitudinal study in children starting at age 7. This will be able to test the ability of the model to capture bone shape changes with growth and capture a more diverse population. Finally, the shape model created for this study and future musculoskeletal model prediction will be available on the opensource platform SimTK.org (https://simtk.org/projects/paed_ssm).

## Conclusions

This work has shown that morphological variation of typically developed paediatric bone can be captured in a statistical shape model. Unknown bone geometries can be predicted to 1.85–2.91 mm accuracy using only demographic and linear bone measurements with performance superior to linear scaling of bone geometry. This statistical shape model provides an accurate and repeatable method for predicting lower limb bone geometry in typically developed children aged 4–18 years and provides a foundation for creating lower limb paediatric musculoskeletal models. This dataset will also be useful to the clinical community to quantify bone morphology across a large typically developed paediatric cohort.

## Supplementary Information

Supplementary Information.

## References

[1] Loder RT, Skopelja EN (2011). The epidemiology and demographics of slipped capital femoral epiphysis. ISRN Orthop..

[2] Kainz H, Wesseling M, Jonkers I (2021). Generic scaled versus subject-specific models for the calculation of musculoskeletal loading in cerebral palsy gait: Effect of personalized musculoskeletal geometry outweighs the effect of personalized neural control. Clin. Biomech..

[3] Schwartz MH, Rozumalski A, Truong W, Novacheck TF (2013). Predicting the outcome of intramuscular psoas lengthening in children with cerebral palsy using preoperative gait data and the random forest algorithm. Gait Posture.

[4] Delp SL (2007). OpenSim: Open-source software to create and analyze dynamic simulations of movement. IEEE Trans. Biomed. Eng..

[5] Davico G (2019). Best methods and data to reconstruct paediatric lower limb bones for musculoskeletal modelling. Biomech. Model. Mechanobiol..

[6] Lebiedowska MK, Polisiakiewicz A (1997). Changes in the lower leg moment of inertia due to child’s growth. J. Biomech..

[7] Beutel BG, Girdler SJ, Collins JA, Otsuka NY, Chu A (2018). Characterization of proximal femoral anatomy in the skeletally-immature patient. J. Child. Orthop..

[8] Gerus P (2013). Subject-specific knee joint geometry improves predictions of medial tibiofemoral contact forces. J. Biomech..

[9] Correa TA, Baker R, Kerr Graham H, Pandy MG (2011). Accuracy of generic musculoskeletal models in predicting the functional roles of muscles in human gait. J. Biomech..

[10] Hainisch R, Kranzl A, Lin YC, Pandy MG, Gfoehler M (2020). A generic musculoskeletal model of the juvenile lower limb for biomechanical analyses of gait. Comput. Methods Biomech. Biomed. Eng..

[11] Hicks JL, Uchida TK, Seth A, Rajagopal A, Delp SL (2014). Is my model good enough? Best practices for verification and validation of musculoskeletal models and simulations of movement. J. Biomech. Eng..

[12] Savonnet L, Duprey S, Jan SVS, Wang X (2019). Pelvis and femur shape prediction using principal component analysis for body model on seat comfort assessment. Impact on the prediction of the used palpable anatomical landmarks as predictors. PLoS ONE.

[13] Ahrend MD (2020). Development of generic Asian pelvic bone models using CT-based 3D statistical modelling. J. Orthop. Transl..

[14] Zhang J, Hislop-Jambrich J, Besier TF (2016). Predictive statistical models of baseline variations in 3-D femoral cortex morphology. Med. Eng. Phys..

[15] Zhang J, Besier TF (2017). Accuracy of femur reconstruction from sparse geometric data using a statistical shape model. Comput. Methods Biomech. Biomed. Eng..

[16] Bryan R, Nair PB, Taylor M (2009). Use of a statistical model of the whole femur in a large scale, multi-model study of femoral neck fracture risk. J. Biomech..

[17] Zhang J, Malcolm D, Hislop-Jambrich J, Thomas CDL, Nielsen PMF (2014). An anatomical region-based statistical shape model of the human femur. Comput. Methods Biomech. Biomed. Eng. Imaging Vis..

[18] Bah MT (2015). Exploring inter-subject anatomic variability using a population of patient-specific femurs and a statistical shape and intensity model. Med. Eng. Phys..

[19] Bryan R, Surya Mohan P, Hopkins A, Galloway F, Taylor M, Nair PB (2010). Statistical modelling of the whole human femur incorporating geometric and material properties. Med. Eng. Phys..

[20] Nolte D, Tsang CK, Zhang KY, Ding Z, Kedgley AE, Bull AMJ (2016). Non-linear scaling of a musculoskeletal model of the lower limb using statistical shape models. J. Biomech..

[21] Baldwin MA, Langenderfer JE, Rullkoetter PJ, Laz PJ (2009). Development of subject-specific and statistical shape models of the knee using an efficient segmentation and mesh-morphing approach. Comput. Methods Prog. Biomed..

[22] Zhang J, Fernandez J, Hislop-Jambrich J, Besier TF (2016). Lower limb estimation from sparse landmarks using an articulated shape model. J. Biomech..

[23] Nolte D, Ko ST, Bull AMJ, Kedgley AE (2020). Reconstruction of the lower limb bones from digitised anatomical landmarks using statistical shape modelling. Gait Posture.

[24] Cignoni, P. *et al*. MeshLab: An open-source mesh processing tool. In *6th Eurographics Ital. Chapter Conf. 2008—Proc.*, 129–136. 10.2312/LocalChapterEvents/ItalChap/ItalianChapConf2008/129-136 (2008).

[25] Jolliffe IT (2002). Principal Component Analysis.

[26] Reyneke CJF, Luthi M, Burdin V, Douglas TS, Vetter T, Mutsvangwa TEM (2019). Review of 2-D/3-D reconstruction using statistical shape and intensity models and X-ray image synthesis: Toward a unified framework. IEEE Rev. Biomed. Eng..

[27] GPL Software. *CloudCompare (v2.10.2)*. http://www.cloudcompare.org/ Accessed 1 June 2020. (2019).

[28] Quintens L (2019). Anatomical variation of the Tibia—A principal component analysis. Sci. Rep..

[29] Herring JA, Tachdjia MO (2020). Tachdjian’s Pediatric Orthopaedics: From the Texas Scottish Rite Hospital for Children.

[30] Novotny SA, Warren GL, Hamrick MW (2015). Aging and the muscle-bone relationship. Physiology.

[31] Bahl JS (2019). Statistical shape modelling versus linear scaling: Effects on predictions of hip joint centre location and muscle moment arms in people with hip osteoarthritis. J. Biomech..

[32] Zhang, J. *et al.* The MAP client: User-friendly musculoskeletal modelling workflows. In *Lect. Notes Comput. Sci. (including Subser. Lect. Notes Artif. Intell. Lect. Notes Bioinformatics)*, Vol. 8789, 182–192. 10.1007/978-3-319-12057-7_21 (2014).

