# Supplementary material for: Morphological variation in paediatric lower limb bones
Source: Sci Rep. 2022 Feb 28;12:3251. doi: 10.1038/s41598-022-07267-4 (PMC8885755; doi:10.1038/s41598-022-07267-4)
Supplement: Supplementary file 1 — Supplementary Information. [file 41598_2022_7267_MOESM1_ESM.docx]

**Morphological Variation in Paediatric Lower Limb Bones – Supplementary Material**

Laura Carman^1^, Thor Besier^1, 2^, Julie Choisne^1,*^

^1^Auckland Bioengineering Institute, The University of Auckland, Auckland, New Zealand

^2^Department of Engineering Science, The University of Auckland, Auckland, New Zealand

*j.choisne@auckland.ac.nz

1. **Deep Segmentation Results**

On the accuracy of the deep segmentation, this was tested using 92 of the manually segmented cases. These cases were divided using 80% for training, 10% for testing, and 10% for validation of the deep segmentation. The results of the testing are shown in Table 2. The totals for the segmented bones were: Pelvis: 331, Femur: 663, and Tibia/Fibula: 659.

Table 1: Deep segmentation testing results showing the Dice score and RMS distance for each bone.

| Bone | Dice Score | RMS Distance (mm) |
| --- | --- | --- |
| Pelvis Left Hemi | 0.94 | 1.35 |
| Pelvis Right Hemi | 0.93 | 1.44 |
| Sacrum | 0.89 | 1.69 |
| Left Femur | 0.95 | 2.53 |
| Right Femur | 0.95 | 1.82 |
| Left Tibia | 0.94 | 2.2 |
| Right Tibia | 0.93 | 5.67 |
| Left fibula | 0.88 | 1.56 |
| Right fibula | 0.83 | 9.19 |

1. **Standard Shape Model Principal Components**
2. *Pelvis*

The first principal component represents the general size scaling and the changes in the ASIS’ and acetabula. The second principal component for the pelvis represents the position of the two ASIS’ and PSIS’ or the rotation of the pelvis. The changes in the back of the acetabulum and the pubic arches are also captured. The third principal component of the pelvis represents the width and the definition of the PSIS’.

1. *Femur*

The first principal component accounts mostly for the size variation in the dataset. However, the growth-related changes are also evident in areas of the greater and lesser trochanter, epicondyles, and condyles of the femur. The second principal component captures the variation in the girth of the femur, shown as an increase in width. The change in neck shaft angle can also be seen in this PC. The third principal component shows the change in anteversion angle and the development of the lesser trochanter.

1. *Tibia/Fibula*

The first principal component accounts mostly for the size variation in the dataset. However, the growth-related changes are also evident in areas of the tibial tuberosity, intercondylar tibial tubercles, the fibular notch, and the proximal tibiofibular joint. The second principal component represents the change in girth. The relationship between the tibia and fibula is also captured in this principal component, at +2SD the fibula bends away from the tibia and at -2SD the fibula bends towards the tibia. The third principal component represents the size changes in the head and lateral malleolus of the fibula. Changes can also be seen in the size of the condyles of the tibia.


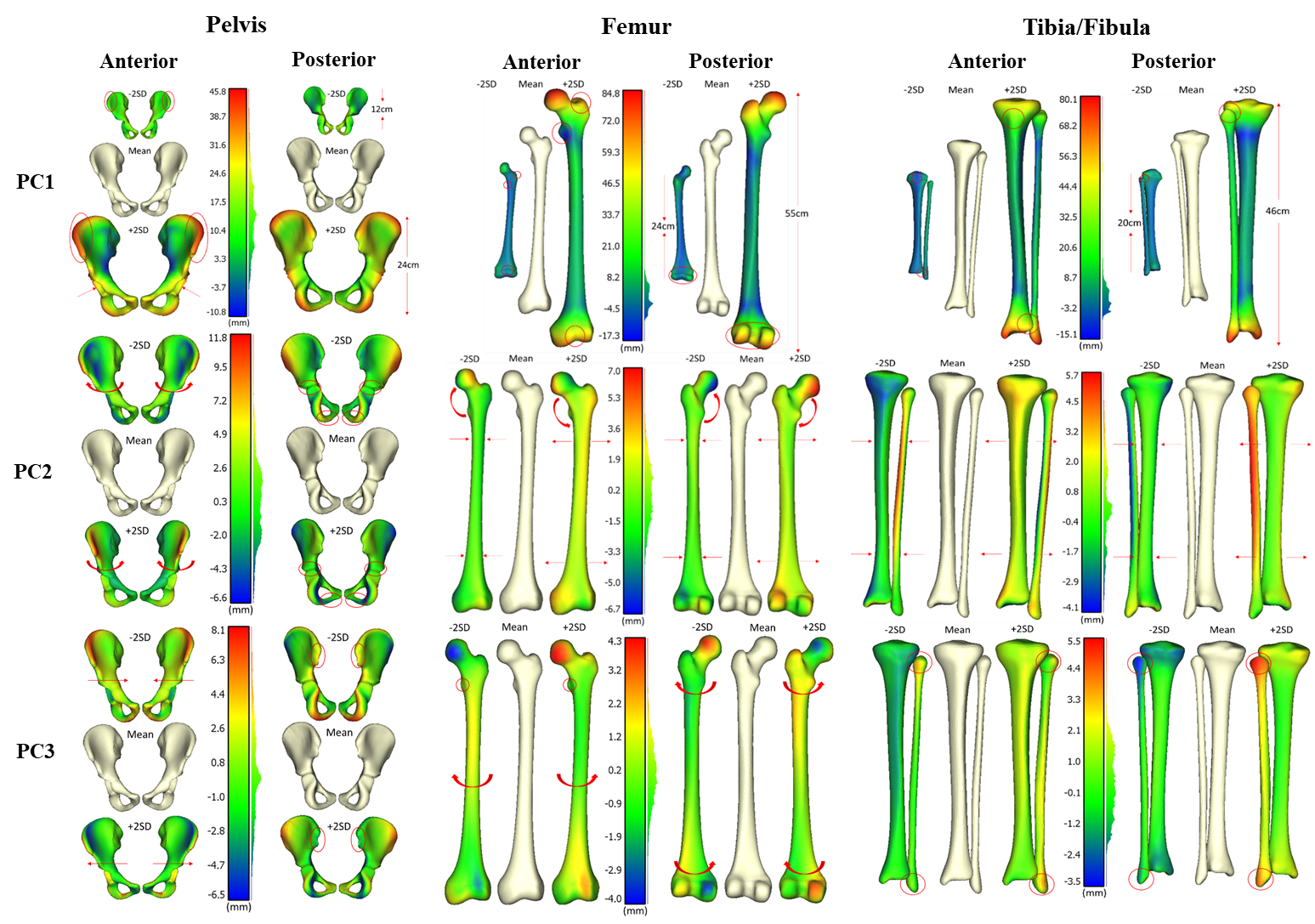


Figure 1: Coloured distance differences of mean meshes (cream) and +/-2SD for first three principal components of the principal component analysis (captures shape and size variation). Anterior (left) and posterior (right) viewpoints are shown and red ellipses and red arrows highlight main features of difference to the mean mesh

1. **Compactness of Shape Model**


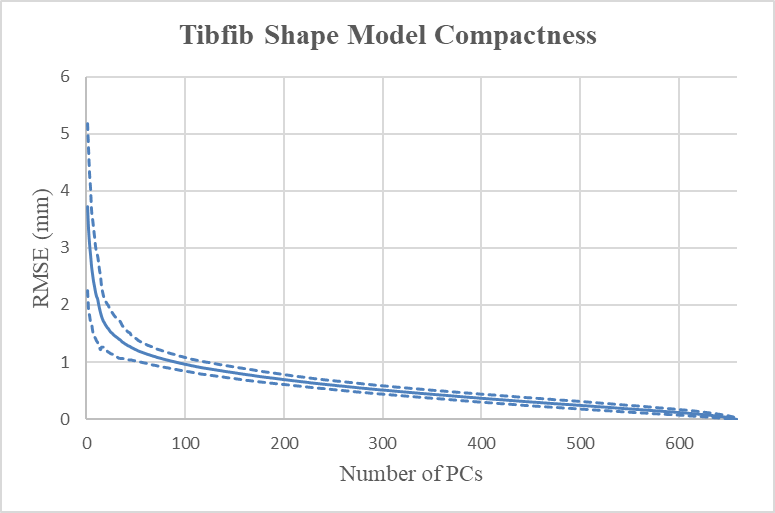

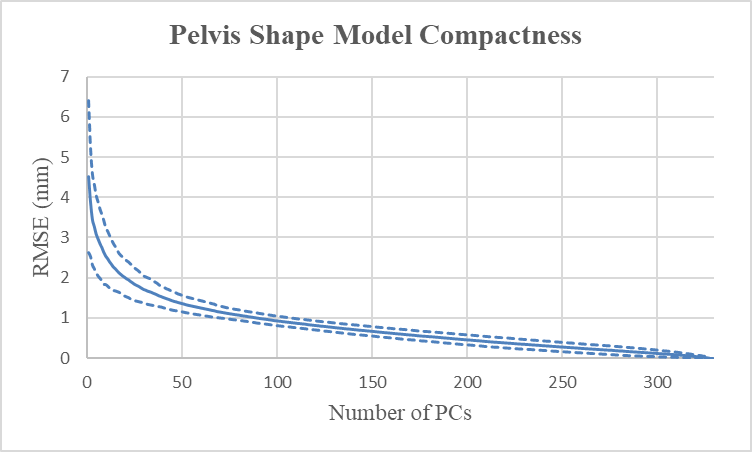

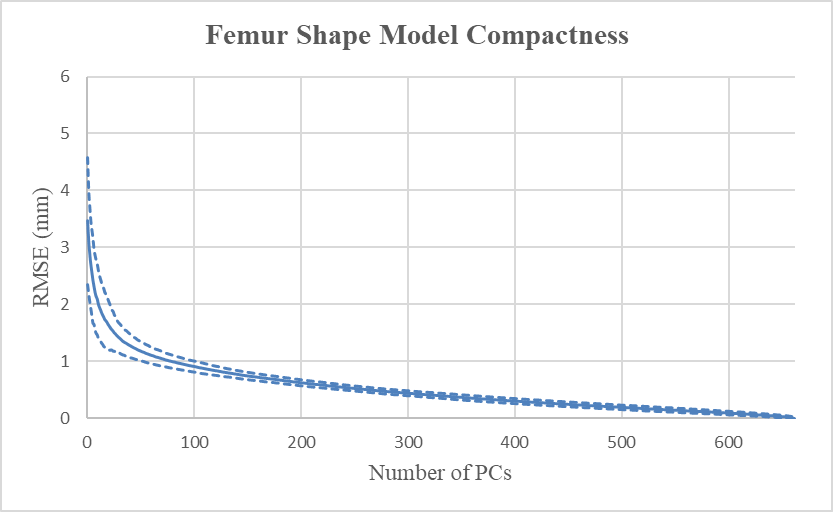


Figure 2: Compactness of the shape model displaying the root mean squared prediction error of the pelvis (top), femur (middle), and tibia/fibula (bottom) for increasing the number of principal components used for prediction in the standard shape model.

1. **Linear scaling comparison**

Table 2: Results showing the average root mean squared error (mm) ± 1SD, and dice score ± 1SD to compare the effect of linear scaling. Comparing the true bone geometry and bone geometry predicted using the shape model, linearly scaled paediatric bone geometry using the mean mesh of the shape model and length measurements, and linearly scaled adult bone geometry using OpenSim geometry and length measurements. This was performed for the Pelvis, Femur, and Tibia/Fibula.

|  |  | **Shape model** | **Linear scaling mean** | **Linear scaling adult** |
| --- | --- | --- | --- | --- |
| **RMSE (mm)** | **Pelvis** | 2.91 ± 0.99 | 4.24 ± 1.77 | 4.79 ± 1.39 |
|  | **Femur** | 2.01 ± 0.62 | 2.37 ± 0.63 | 4.38 ± 0.72 |
|  | **Tibia/Fibula** | 1.85 ± 0.54 | 2.36 ± 0.58 | 4.39 ± 0.86 |
| **Dice Score** | **Pelvis** | 0.77 ± 0.07 | 0.66 ± 0.14 | 0.56 ± 0.12 |
|  | **Femur** | 0.89 ± 0.03 | 0.87 ± 0.04 | 0.69 ± 0.05 |
|  | **Tibia/Fibula** | 0.86 ± 0.04 | 0.82 ± 0.07 | 0.70 ± 0.04 |


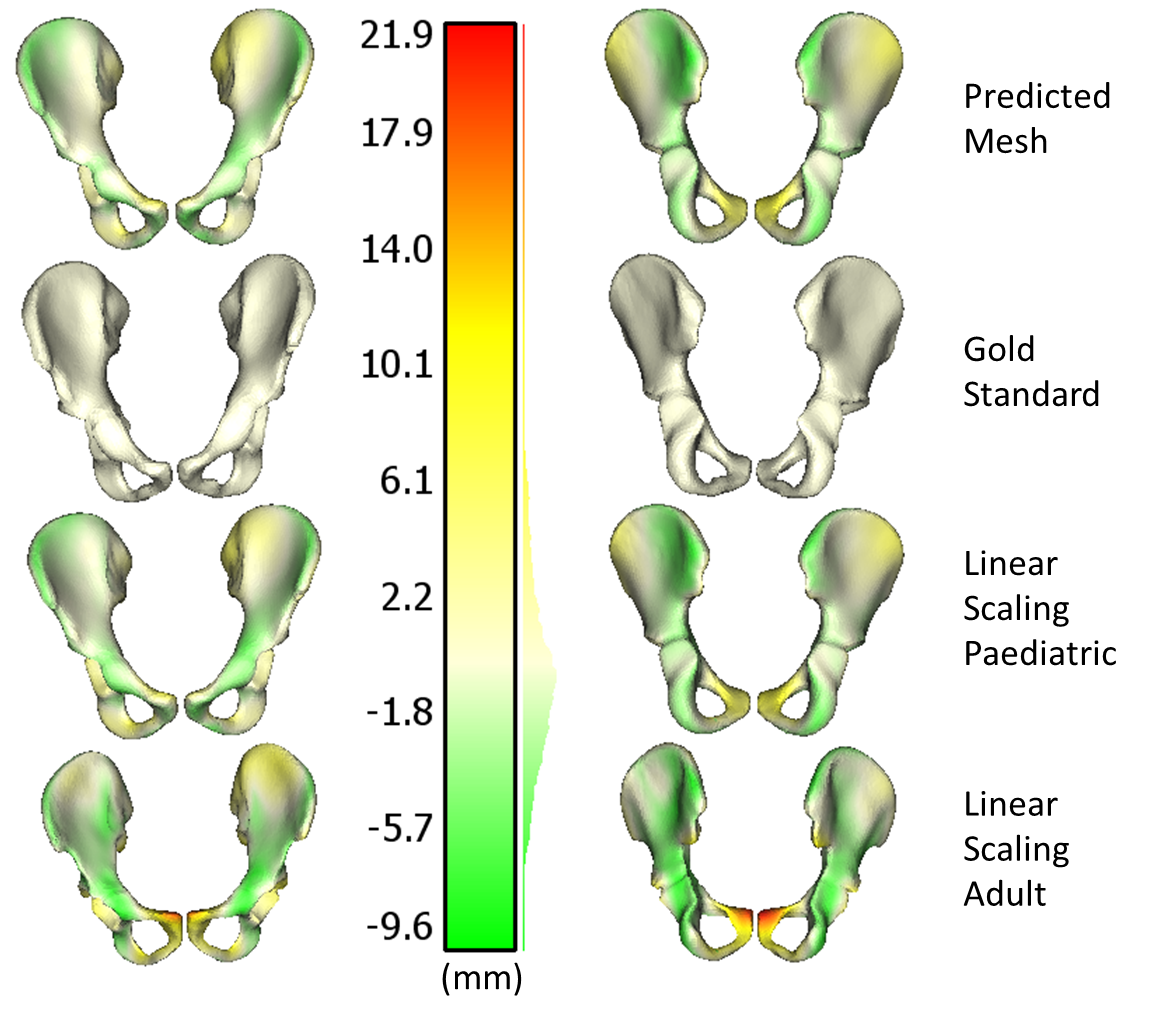

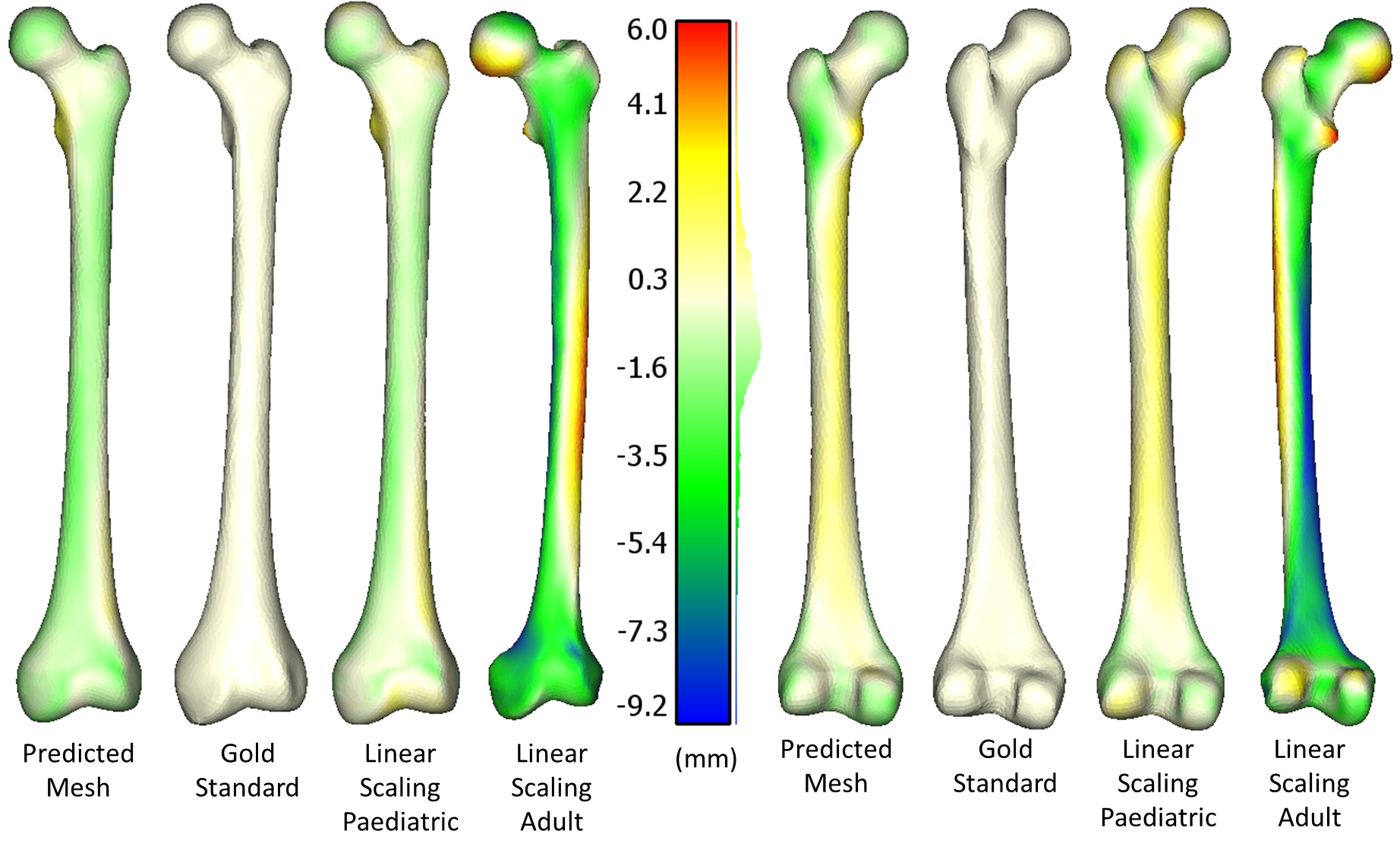

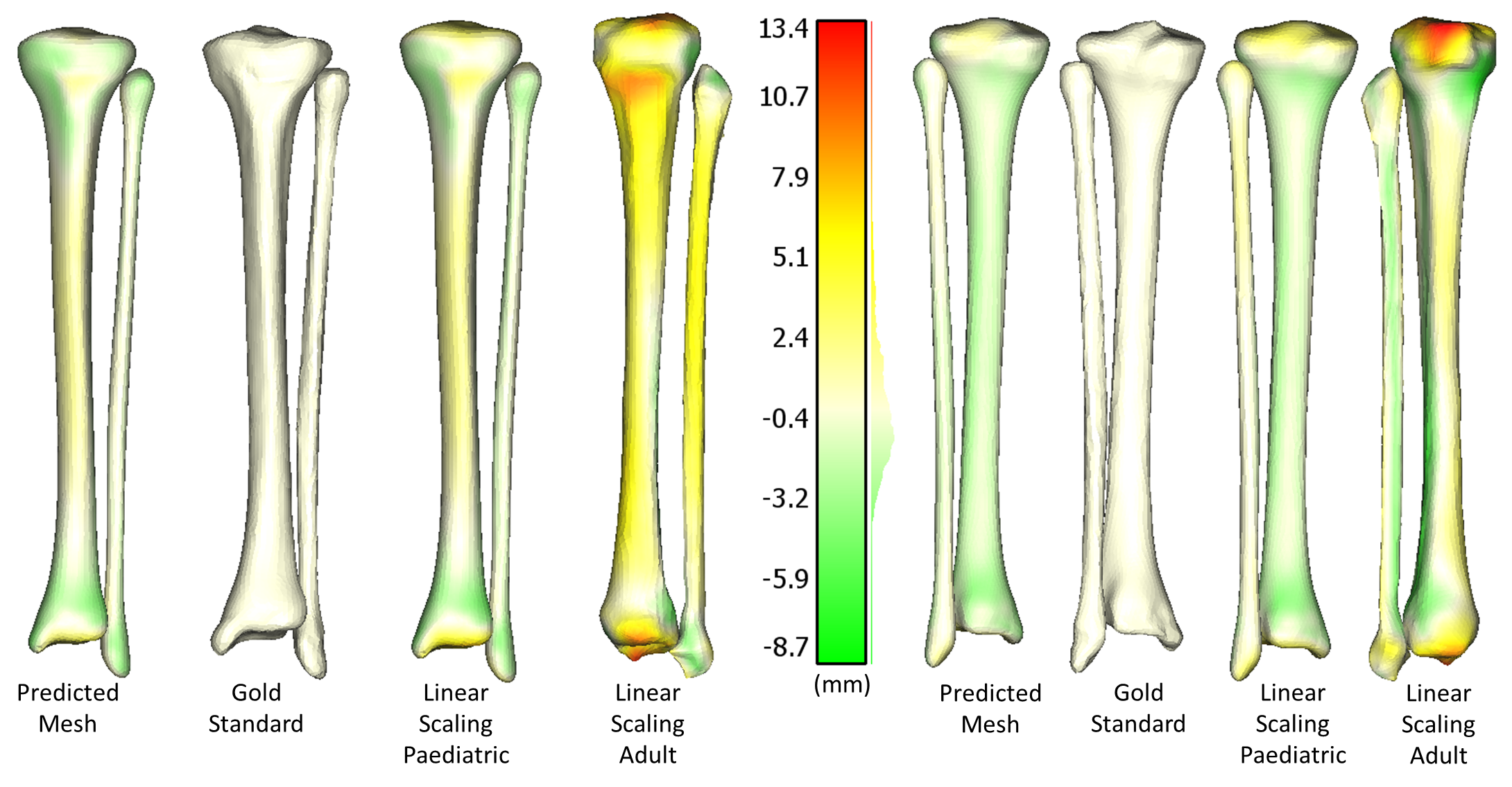


**Pelvis**

**Femur**

**Tibia/**

**Fibula**

Figure 3: Example of coloured distance differences in bone geometry from one average case (Male, 11 years, 145cm, 41kg) showing “gold standard” geometry in cream, “predicted” geometry from the shape model, “linear scaling paediatric” bone geometry of the mean mesh in the dataset, and “linear scaling adult” bone geometry from Opensim.

Figure 4: Example of coloured distance differences in bone geometry from highest errors in adult linear scaling for each bone showing “gold standard” geometry in cream, “predicted” geometry from the shape model, “linear scaling paediatric” bone geometry of the mean mesh in the dataset, and “linear scaling adult” bone geometry from Opensim.


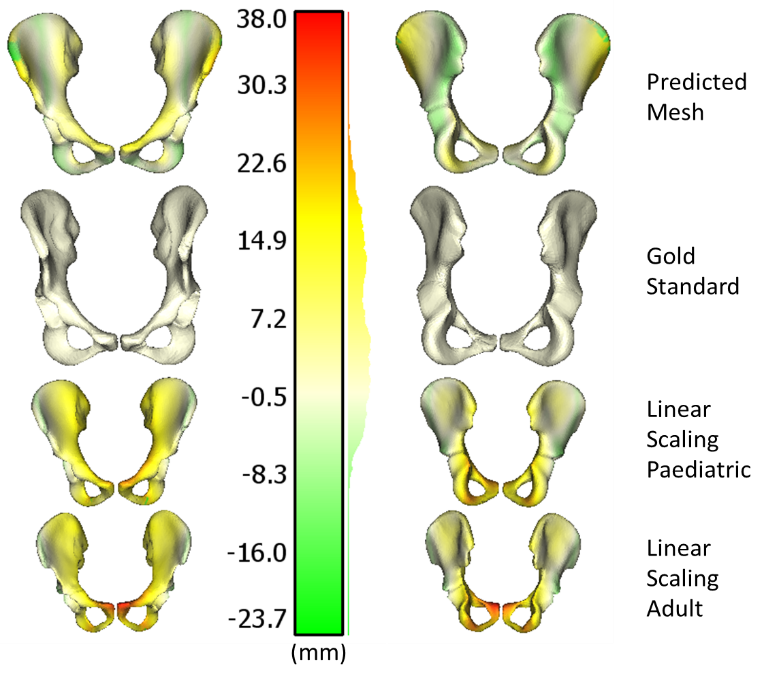

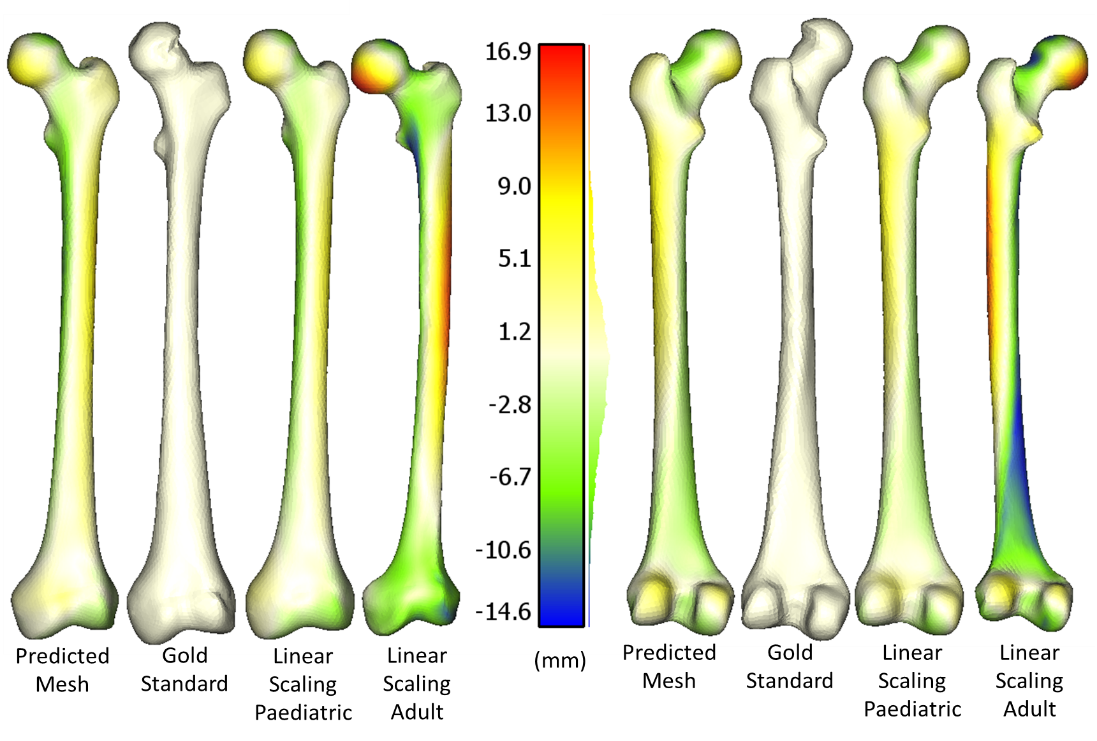

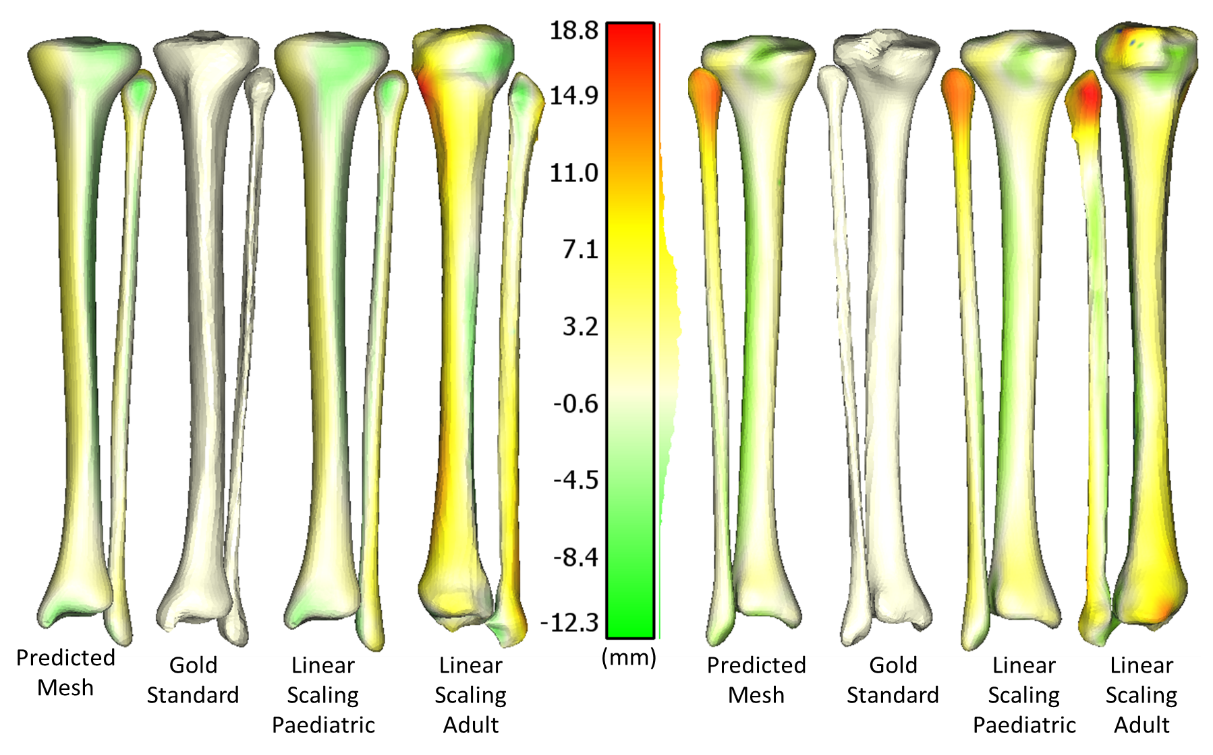


**Pelvis**

**Femur**

**Tibia/**

**Fibula**
